# Childhood Leukemia Survivors and Metabolic Response to Exercise: A Pilot Controlled Study

**DOI:** 10.3390/jcm9020562

**Published:** 2020-02-19

**Authors:** Charline Pegon, Emmanuelle Rochette, Nadège Rouel, Bruno Pereira, Eric Doré, Florentina Isfan, Victoria Grèze, Etienne Merlin, Justyna Kanold, Pascale Duché, on behalf of HERCCULE Group

**Affiliations:** 1 CHU Clermont-Ferrand, Pédiatrie, F-63000 Clermont-Ferrand, France; cpegon@chu-clermontferrand.fr (C.P.); nrabiau@chu-clermontferrand.fr (N.R.); edore@chu-clermontferrand.fr (E.D.); fisfan@chu-clermontferrand.fr (F.I.); vgreze@chu-clermontferrand.fr (V.G.); e_merlin@chu-clermontferrand.fr (E.M.); jkanold@chu-clermontferrand.fr (J.K.); 2 Université Clermont Auvergne, INSERM, CIC 1405, Unité CRECHE, F-63000 Clermont-Ferrand, France; 3 Université de Toulon, Laboratoire IAPS, F-83041 Toulon, France; pascale.duche@univ-tln.fr; 4 CHU Clermont-Ferrand, Délégation de la Recherche Clinique et Innovations, F-63000 Clermont-Ferrand, France; bpereira@chu-clermontferrand.fr; 5 Université Clermont Auvergne, INRA, UMR 1019 UNH, ECREIN, F-63000 Clermont-Ferrand, France; 6 Université Clermont Auvergne, Laboratoire des Adaptations Métaboliques en conditions Physiologiques et Physiopathologiques (AME2P), EA 3533, F-63000 Clermont-Ferrand, France

**Keywords:** acute leukemia, children, metabolism, fat oxidation, physical fitness

## Abstract

Background: Leukemia is the most common cancer in pediatrics, with many late effects such as higher risk of dyslipidemia, insulin resistance, obesity, and metabolic syndrome. The objective of this work was to investigate substrate oxidation during submaximal exercise in survivors of childhood acute leukemia. Methods: A total of 20 leukemia survivors and 20 healthy children were matched by sex, age, and Tanner stage. They all took a submaximal incremental exercise test to determine fat and carbohydrate oxidation rates. Results: Cardiorespiratory fitness was significantly lower in leukemia survivors, with lower relative VO_2_ peaks (*p* < 0.001), lower heart rate values (*p* = 0.02), and lower exercise power (*p* = 0.012), whereas rest metabolism and body mass index did not differ between the two groups. During exercise, upward of heart rate relative to VO_2_ peak was significantly higher (*p* < 0.001) in childhood leukemia survivors. We found lower carbohydrate and fat oxidation rates (*p* = 0.07) in leukemia survivors compared with healthy children, and also a significantly lower relative maximal fat oxidation rate (*p* = 0.014). Conclusion: Despite impaired physical fitness and metabolic response to exercise, childhood leukemia survivors remained sensitive to physical activity interventions, and could readily adapt to submaximal exercise intensity.

## 1. Introduction

Acute childhood leukemia, lymphoblastic (ALL) and myeloblastic (AML), is the most common cancer in pediatrics, with an overall age-specific incidence of 46.4 per million persons per year [1]. Although prevalence has been increasing in recent decades, survival rates have also improved, from 65% to 85% depending on the type of leukemia [2,3,4]. The long-term health condition of such patients is still a major public health concern. Leukemia treatments, which always depend on several chemotherapies and sometimes radiotherapy and bone marrow transplantation, have significantly improved the prognosis of these children. Even so, treatment leaves 50% of them with at least one chronic medical disease that has a significant impact on mortality and morbidity [5]. Several articles have been published on late effects of treatment in this population, notably increased endocrine deficiency [6,7], obesity [8], mitochondrial dysfunction [9], pulmonary [10] and neuromuscular [11] toxicity, cardiovascular diseases [12], and metabolic syndrome [13,14]. This metabolic syndrome is related to a range of disorders including insulin resistance, central obesity, high blood glucose, dyslipidemia, and high blood pressure, and is an important risk factor for cardiovascular disease [15].

Long-term cardiometabolic diseases and metabolic syndrome in leukemia survivors may cause metabolic impairments [16]. The ability to oxidize lipids and carbohydrates during incremental exercise reflects a profile of metabolic fitness correlated to the physiological status of the muscles [17,18]. In children cured of acute leukemia, despite advances in diagnostic and therapeutic methods, levels of physical activity deteriorate [19], and there is an increase in sedentary lifestyle [20] and fatigue [21] closely linked to excess energy intake and reduced energy expenditure [22]. Survivors of childhood leukemia have increased incidence of metabolic syndrome and impaired muscular [23] and cardiorespiratory functional capacity [24,25] that may reflect impaired metabolism and substrate utilization during exercise compared to their healthy counterparts. The published evidence is positive for the impact of exercise on muscle strength and flexibility, and is mixed for the impact of physical activity intervention on cardiopulmonary fitness among children with an acute lymphoblastic leukemia and among survivors exposed to cardiotoxic agents [26].

Our aim was to determine whether the disease and its treatment affected metabolic responses to exercise. For this purpose, we studied substrate oxidation during submaximal exercise in leukemia survivors compared to matched healthy controls.

## 2. Methods

### 2.1. Participants

This prospective cross-sectional case-control study ran from May 2019 to October 2019. It included children cured of acute leukemia followed at Clermont-Ferrand University Hospital in France and their matched healthy controls. Children in remission of leukemia of any type (ALL and AML), aged 6–18 years, without treatment for at least one year and without physical disability due to the disease were compared to an equal number of healthy controls recruited as peers and matched for age, sex, and sexual maturity. Physicians assessed sexual maturity using pubic hair indices and male genital or female breast development, as described by Tanner [27]. Subjects were excluded if they had an infection diagnosed by a physician, if they had corticosteroid treatment in the previous three months, if they were unable to exercise, or if they had a contraindication to physical exercise (cardiorespiratory disorders incompatible with submaximal exercise or orthopedic or neurologic dysfunctions that ruled out pedaling). We firstly included leukemia survivors, then we included matched healthy controls. Healthy controls were recruited from the entourage (classmates, family, sports club) of the patients.

This study was carried out in accordance with the recommendations of Comité de Protection des Personnes (CPP) Ile-de-France VII, (no. 2018-A03247-48) with written informed consent from all subjects, as prescribed by the Declaration of Helsinki. The protocol was approved by the Comité de Protection des Personnes (CPP) Ile-de-France VII. Trial registration: in ClinicalTrials.gov, reference number NCT 03913962, registered on 12 April 2019.

### 2.2. Physical Activity Assessment

To assess physical activity level (PAL), we asked the children, with the help of their parents, to keep an activity booklet logging the child’s physical activity five days a week during schooldays and two days on weekends. To assess physical activity levels, we used validated a pediatric questionnaire—the International Physical Activity Questionnaire for Adolescents (IPAQ-A) [28]. Total metabolic equivalent of task (MET)-minutes per week was calculated using the formula: [walking MET-minutes/week = 3.3 × walking minutes × walking days] + [moderate MET-minutes/week = 4.0 × moderate-intensity activity minutes × moderate days] + [vigorous MET-minutes/week = 8.0 × vigorous-intensity activity minutes × vigorous-intensity days] = total physical activity (PA) MET-minutes/week [29].

### 2.3. Experimental Procedure

Submaximal exercise was performed at least 3 h after the last meal. The children avoided calorie-rich food and refrained from strenuous physical activity for at least 24 h beforehand. After sitting for 20 min (rest metabolism was measured during the last 10 min with oxygen consumption) [30], the subjects performed, to the point of volitional fatigue, a graded submaximal exercise test on an electromagnetically braked cycle ergometer with continuous gas collection and heart rate monitoring. Following a 2 min warm-up consisting of unloaded pedaling, subjects at Tanner stages 1 and 2 started cycling at 10 W, and their work rate was increased by 10 W every 3 min. Subjects at Tanner stages greater than or equal to 3 started at 20 W, and their work rate was increased by 15 W every 3 min. When heart rate was unstable (heart rate variation more than ±5 beats per minute), this stage was extended for up to 5 min to obtain a heart rate stable to within ±5 beats. When the respiratory exchange ratio (RER) was greater than or equal to 1.00 (indicating the absence of fat oxidation), work rate was increased by the same increments at 1 min intervals until volitional fatigue was reached. The VO_2_ peak was considered to have been reached when the RER was greater than or equal to 1.05 and the subject achieved his or her age-predicted maximal heart rate (HRmax: 220—age), according to the methodology validated by Riddell et al. [31].

### 2.4. Measurements

All the tests were performed on a Cyclus 2 ergometer (RBM Elektronik-Automation GmbH, Leipzig, Germany). Oxygen consumption (VO_2_) and carbon dioxide (VCO_2_) were measured breath by breath through a mask connected to an O_2_ and CO_2_ analyzer (MetaMax 3b, Cortex Biophysik, Leipzig, Germany). Ventilatory parameters were averaged every minute during the submaximal exercise test and the subsequent 10 min recovery period. Heart rate was monitored continuously throughout the duration of the tests (Polar RS 800cx monitor, Polar, Finland).

### 2.5. Data Analysis

Indirect calorimetry is the recognized standard method of quantifying substrate oxidation rates at rest and during exercise [32]. The intensity of the exercise was calibrated according to theoretical VO_2_max estimated in milliliter per minute (mL/min) from the Wasserman equations [33]: male = (52.8 × weight) – 303.4, female = (28.5 − weight) + 288.1. The VO_2_ and VCO_2_ values were averaged over the last minute of each work rate, and the results used to calculate fat oxidation over a wide range of exercise intensities for each subject using Péronnet and Massicotte’s equation [34]: lipid (mg/min) = 1.6946 × VO_2_ − 1.7012 × VCO_2_, carbohydrate (CHO) (mg/min) = 4.585 × VCO_2_ − 3.2255 × VO_2_.

For each child, a best-fit polynomial curve was constructed for fat and CHO oxidation rate (expressed as milligram per minute) vs. exercise intensity (expressed as a percentage of the VO_2_ peak). Each individual curve was then used to determine the peak fat oxidation rate and the exercise intensity associated with the maximal fat oxidation (MFO) rate [35].

### 2.6. Statistical Considerations

The sample size was determined according to (i) CONSORT 2010 statement, extension to randomized pilot, and feasibility trials [36], and (ii) Cohen’s recommendations [37], which define effect size (ES) bounds as small (ES: 0.2), medium (ES: 0.5), and large (ES: 0.8, “grossly perceptible and therefore large”). With 20 participants per group, an effect size greater than 1 could be evidenced for a two-sided type I error at 5% and a statistical power greater than 80%.

Statistical analyses were performed using Stata software (version 13, StataCorp, College Station, USA). The tests were two-sided with a type I error set at 5%. Continuous data were expressed as mean ± standard deviation (SD) or median (interquartile range) according to statistical distribution. The assumption of normality was assessed using the Shapiro–Wilk test. For non-repeated measures, continuous variables were compared between independent groups using Student’s *t*-test, or the Mann–Whitney test when the *t*-test assumptions were not met. Homoscedasticity was analyzed using the Fisher–Snedecor test. The results were expressed as effect-sizes and 95% confidence intervals. The study of relationships between continuous parameters was analyzed by estimating Pearson or Spearman correlation coefficients (noted as *r*). For measurements at each scale, usual statistical tests were inappropriate because the hypothesis of data independence was not met (several measurements for the same subject). Random effects models for correlated data (more precisely linear mixed model) were also used to take into account between- and within-subject variability. Scale-point evaluations, group, and their interactions were considered as fixed effects, and subject was a random effect. The assumption of residual normality was tested as described above. When appropriate, a logarithmic transformation was used to obtain normality of the dependent variable and guarantee the correct use of the above analyses. Finally, these analyses were completed by a multivariable approach to adjust univariate results on possible confounder covariates determined according to their clinical relevance: age, sex, Tanner stage, and body mass index (BMI) *z*-score. These parameters were added to the random-effects models as fixed effects.

## 3. Results

### 3.1. Patients

**Figure 1:**
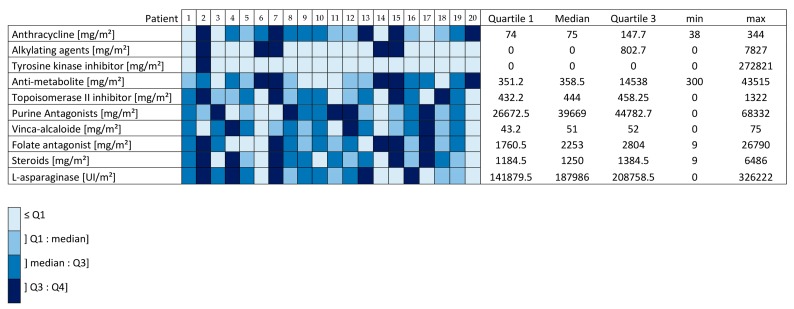
Cumulative chemotherapy heatmap. Alkylating agents: busulfan, cyclophosphamide, melphalan, iphosphamide; thyrosine kinase inhibitor: imatinib; anti-metabolite: cytarabine, amsacrine; topoisomerase II inhibitor: etoposide; purine antagonists: 6-thioguanine, 6-mercaptopurine; vinca-alcaloide: vindesine, vincristine; folate antagonist: methotrexate.

The characteristics of the subjects are summarized in Table 1. Most were children cured of ALL (18 ALL versus 2 AML). Total cumulative dose of chemotherapies is reported in Figure 1. In the whole population, no patients had radiation therapy and two patients had hematopoietic stem cell transplantation. There was no significant difference between our childhood leukemia survivors and healthy children in rest metabolism (1577.9 ± 579.4 vs. 1553.5 ± 517.3 kcal.day^−1^, *p* = 0.51, Hedge’s *g* = −0.04 (−0.66, 0.58)), physical activity level (1883.5 ± 1042.9 vs. 2527.7 ± 1496.2 MET-minutes/week, *p* = 0.30, Hedge’s *g* = 0.49 (−0.14, 1.12)), and in BMI for age percentile (50.5 ± 28.9 vs. 42.3 ± 27.3 percentile, *p* = 0.35, Hedge’s *g* = −0.29 (−0.91, 0.34)). Absolute VO_2_ peak (mL min^−1^) and relative VO_2_ peak (VO_2_ peak kg^−1^ of body weight) were significantly lower in the childhood leukemia survivors than in the control group (respectively 1245.4 ± 413.3 vs. 1603.2 ± 621.9 mL min^−1^, *p* = 0.003, and 29.4 ± 6.9 vs. 40.6 ± 11.3 mL.min^−1^ kg^−1^, *p* < 0.001). Likewise, the measured maximum heart rate during exercise was significantly lower in the childhood leukemia survivors than in the controls (155 ± 17 vs. 168 ± 19 beats min^−1^, *p* = 0.016).

### 3.2. Oxidation of Metabolites

**Figure 2:**
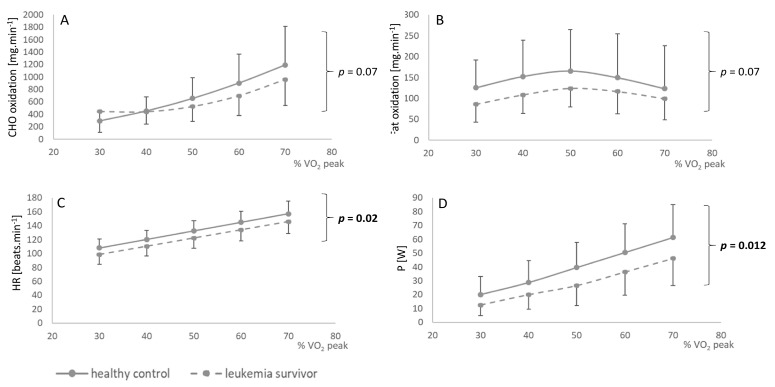
Fat (**A**) and carbohydrate (**B**) oxidation rate, heart rate (**C**), and power (**D**) according to percentage of VO_2_ peak. Data are means ± SD.

**Figure 3:**
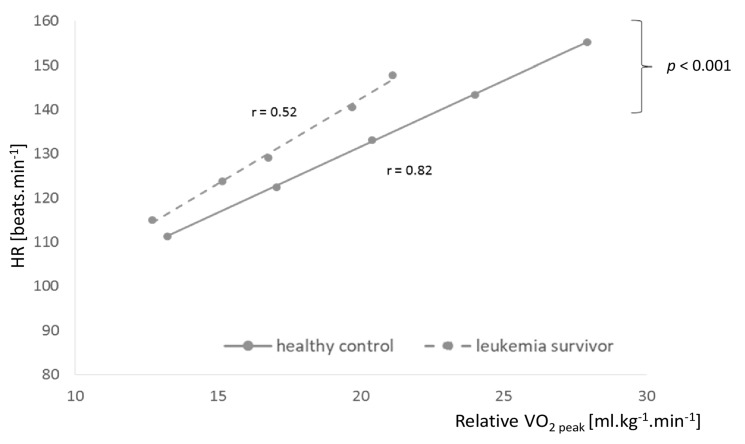
Individual regression for heart rate vs. the corresponding relative VO_2_ peak for leukemia survivors and healthy controls. Data are means ± SD.

The oxidation rates of fat and carbohydrates as a function of percentage of VO_2_ peak are shown in Figure 2. At exercise intensities corresponding to the same percentages of VO_2_ peak, the rates of carbohydrates and fat oxidation were lower in leukemia survivors (*p* = 0.07) (Figure 2A,B). However, for exercise intensities corresponding to the same VO_2_ peak percentages, we found significantly lower heart rate values (*p* = 0.02) and lower exercise power (*p* = 0.012) for childhood leukemia survivors than for controls (Figure 2C,D). Heart rate versus relative VO_2_ peak during exercise was significantly displaced upward and displayed a greater slope (*p* < 0.001), comparing childhood leukemia survivors with healthy controls (Figure 3).

### 3.3. Maximal Fat Oxidation

**Figure 4:**
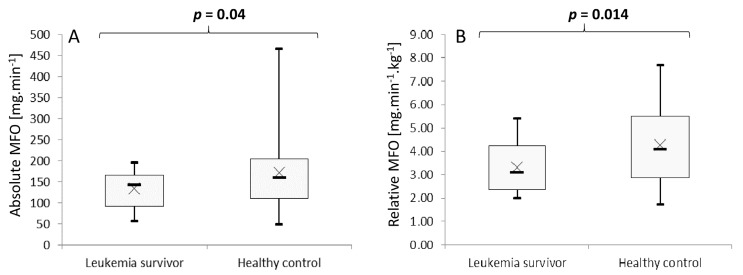
Comparison of absolute maximal fat oxidation (**A**) and relative maximal fat oxidation (**B**). Boxes represent interquartile ranges. Whiskers give minimum and maximum values. Data are means (*×*) and medians (–).

The maximal fat oxidation rate (MFO) was significantly different between the two groups. For childhood leukemia survivors and controls, the respective absolute and relative MFOs were 133.4 ± 43.4 vs. 172.1 ± 96.1 mg min^−1^ (*p* = 0.04) and 3.2 ± 1.1 vs. 4.3 ± 1.8 mg min^−1^ kg^−1^ (*p* = 0.014) (Figure 4A,B).

Between childhood leukemia survivors and controls, there was no difference in heart rate (123 ± 17 vs. 128 ± 20 beats min^−1^, *p* = 0.15, Hedge’s *g* = 0.25 (−0.37, 0.87)) or percentage of VO_2_ peak (51.1 ± 7.7 vs. 47.9 ± 15.2%, *p* = 0.55, Hedge’s *g* = −0.77 (−1.14, −0.12)) or RER (0.88 ± 0.03 vs. 0.87 ± 0.04, *p* = 0.09, Hedge’s *g* = −0.28 (−0.90, 0.35)) to reach MFO. However, power to reach MFO was significantly lower in leukemia survivors than in controls (27.3 ± 11.6 vs. 35.2 ± 16.2 W, *p* = 0.03).

## 4. Discussion

All the children enrolled completed the exercise test with no adverse effects (such as a cardiovascular event, lightheadedness, or general malaise). Submaximal exercise was feasible and safe for children surviving leukemia. In our study, the first main finding was that the carbohydrate and fat oxidation rates, independently of exercise intensity, were lower in leukemia survivors than in healthy controls, also with a significantly lower maximal fat oxidation. Secondly, relative and absolute VO_2_ peaks were significantly lower in leukemia survivors, as were heart rate values during this submaximal exercise. These results suggest a dysregulation of metabolic and physical fitness during exercise in leukemia survivors, although they still adapted well to exercise, with no significant difference between the two groups in terms of heart rate, RER, or percentage of VO_2_ peak to reach MFO.

VO_2_ peak reflects cardiovascular fitness, a determining factor in response to exercise. There were significantly lower absolute and relative VO_2_ peaks in leukemia survivors, associated with significantly lower measured maximum heart rates independently of exercise intensity in these children. This result could reflect an impairment in cardiorespiratory fitness in these patients and could be a side effect of treatments, especially anthracyclines. All the leukemia survivors received anthracyclines (see Figure 1), a drug with high cardiotoxicity, known to adversely affect cardiomyocytes and modify the function of the left ventricle, as well as the ability to perform exercise [38]. An impact on cardiac function was probably identified with significantly lower heart rate values for exercise intensities corresponding to the same percentage of VO_2_ peak in leukemia survivors than in controls. This probable cardiotoxicity was not confirmed by echocardiography, with a normal left ventricular ejection fraction (LVEF 69.2 ± 2.7) in leukemia survivors, which could be explained by the early examination and lack of ultrasound data during the exercise. However, there is evidence that anthracycline cardiotoxicity is dose-related and cumulative, leading to congestive heart failure or asymptomatic left ventricular dysfunction [39]. Hence, lower VO_2_ peaks in leukemia survivors compared with healthy controls could be due to chemotherapy, but also to a lower level of physical activity. In our study, we found no significant difference in physical activity level between these two groups, either with the IPAQ questionnaire or the MET minutes/week calculation. Nevertheless, MET minute/week values showed a medium effect size (1883.5 ± 1042.9 vs. 2527.7 ± 1496.2 MET minutes/week, *p* = 0.30, Hedge’s *g* = 0.49 (−0.14, 1.12)), suggesting that leukemia survivors could be less physically active overall than healthy controls. This would be in line with studies reporting a lower level of physical activity in leukemia survivors [40], particularly in relation to the lack of physical activity throughout the duration of their prolonged treatments (including multiple chemotherapies and repeated hospitalizations) associated with secondary impairment of physical fitness and an increased risk of chronic health problems [41]. In Figure 3, we can see that leukemia survivors always had heart rates higher than controls, with a relative VO_2_ that was lower. This corroborated previous results, namely, a lower VO_2_ peak in leukemia survivors. This VO_2_ peak was considered as an individual index of aerobic fitness, and this graph could be interpreted as showing a higher fatigability when performing physical exercise, even submaximal, which could be related to a probable impairment in cardiorespiratory and physical fitness in these patients.

In our study, there was a significantly lower MFO in leukemia survivors, which could reflect impaired metabolic fitness, especially because they had lower lipid oxidation rates and carbohydrate oxidation rates (see Figure 2), irrespective of exercise intensity. Although these results point to a probable dysfunction in metabolic abilities in these patients during a submaximal exercise, they still adapted well to the exercise, with no significant difference between the two groups in heart rate, RER, or percentage of VO_2_ peak to reach MFO. These values reflect the intensity at which maximal rates of fat oxidation occur during exercise, also called Fatmax. MFO and Fatmax can vary independently, especially according to interindividual variations (sex, body composition, physical activity level) [42]. In our study, this lower MFO associated with a lower power at MFO could indicate a metabolic impairment and could be explained by a lessened oxidative capacity of muscle during exercise. Skeletal muscle is the main site where exercise adaptation reactions occur in terms of metabolic flexibility, in close interaction with mitochondrial signaling pathways [43]. Metabolic flexibility could therefore be disturbed by impaired mitochondrial function in these patients, as described in the literature, with degraded beta-oxidation [9], but could also be secondary to impaired absorption, transport, and oxidation of energy-rich substrates in skeletal muscle. These hypotheses could explain our results and suggest that the substrate-metabolism impairment is at the muscle level. The impact of treatments on muscle function therefore concerns both the neuromuscular level and the level of the oxidative function. Multiple chemotherapies can lead to dysfunctional organ systems, particularly musculoskeletal, cardiac for anthracyclines, and pulmonary for cyclophosphamide [10] and methotrexate [44], which can also increase the risk of a limitation of physical performance. Studies have also shown that treatment with vincristine has repercussions on the motor neuron control activity or on the depletion of muscle motor units, which may lead to decreased motor abilities [11], probably responsible for a weaker development of their muscle density. Muscle changes in cancer patients include significant deficiencies and loss of muscle mass that can lead to chronic muscle weakness [45], which seems to be related to physical deconditioning [46]. This could also explain why exercise power, independently of intensity, was lower for this population than for controls. This lower power would therefore seem to be related to an impairment of muscle function, especially in terms of muscle performance, and not in terms of muscle mass, because there was no significant difference between these two groups in the rest metabolism (1577.9 ± 579.4 vs. 1553.5 ± 517.3 kcal day^−1^, *p* = 0.51, Hedge’s *g* = −0.04 (−0.66, 0.58)) and body mass index for age percentile (50.5 ± 28.9 vs. 42.3 ± 27.3 percentile, *p* = 0.35, Hedge’s *g* = −0.29 (−0.91, 0.34)). In addition, Lanfranconi et al. [47] reported an impairment of muscle O_2_ extraction ability during exercise in leukemia survivors, probably secondary to impaired skeletal muscle oxidative function. This limitation in oxidative muscle extraction could be explained by impaired muscle function due to the catabolic effect of treatments, notably vincristine and corticosteroids, which may lead to a limitation of the muscle fibers’ ability to consume O_2_. Hence, the skeletal muscle oxidative system could be degraded in leukemia survivors, an effect aggravated by a low level of physical activity and sedentary habits [48], causing impairment of metabolic fitness.

A larger study would be useful to evaluate muscle mass and function. Data need to be extended with body composition (absorptiometry), the metabolic status of these patients (dyslipidemia blood test), and an exploration of their respiratory function (spirometry test). In addition, these children performed a submaximal test, resulting in a maximum heart rate not reached in the leukemia survivors when compared with the controls. It would also be useful to assess the patient’s perception after performing this type of submaximal exercise to determine whether there is lower exercise tolerance in these patients.

## 5. Conclusions

The present study showed an impairment in physical fitness (with lower relative and absolute VO_2_ peak) and also in metabolic fitness (with lower MFO and lower substrate oxidation during submaximal exercise) in leukemia survivors, although they remained well adapted to exercise, with no difference in exercise intensities at which MFO was achieved. In our study, the leukemia survivors were not overweight, a common late effect in this population, and they also had lower levels of physical activity. In the long term, there is a cumulative effect of treatment-related undesirable effects with an increase in weight associated with insufficient physical activity that could further damage cardiorespiratory adaptation and metabolic abilities of these patients. This could result in greater fatigability and poorer adaptation, even for low intensity exercise. An important point is that the leukemia survivors remained sensitive to physical activity interventions, and thus the prescription of physical activity adapted to their cardiorespiratory and muscular fitness could allow greater tolerance to exercise and therein improve physical and metabolic fitness. Finally, it is manifestly essential to encourage the practice of regular exercise in this population to reduce physical and metabolic impairments and improve quality of life by limiting long-term cancer complications.

## Figures and Tables

**Table 1:** Participants’ characteristics.

	AL Survivors	Healthy Controls	*p*	Hedge’s *g*
*n*	20	20		
Sex (male/female)	10/10	10/10		
Age (years) mean ± SD	12.2 ± 3.3	12.3 ± 3.4	ns	
Tanner stage (I–II/III–V)	13/7	13/7		
Body mass index for age percentile, mean ± SD	50.5 ± 28.9	42.3 ± 27.3	ns	−0.29 (−0.91, 0.34)
BMI (kg/m²) mean ± SD	19.0 ± 3.7	17.8 ± 2.9	ns	
Absolute VO_2_ peak (mL/min) mean ± SD	1245.4 ± 413.3	1603.2 ± 621.9	0.003	
Relative VO_2_ peak (mL/kg/min) mean ± SD	29.4 ± 6.9	40.6 ± 11.3	<0.001	
Rest metabolism (kcal/day) mean ± SD	1577.9 ± 579.4	1553.5 ± 517.3	ns	−0.04 (−0.66, 0.58)
Exercise maximum heart rate (bpm) mean ± SD	155.4 ± 17.0	168.1.7 ± 19.3	0.016	
Level of physical activity (MET-minutes/week) mean ± SD	1883.5 ± 1042.9	2527.7 ± 1496.2	ns	0.49 (−0.14, 1.12)
Age at diagnosis (years), mean ± SD	4.8 ± 3.4	NA		
Time since end of treatment (years) mean ± SD	4.9 ± 3.5	NA		
Type of acute leukemia (*n*)	20	NA		
*AL lymphoblastic*	18			
*Leukocytosis < 50,000/mm^3^ + no MI*	15			
*Leukocytosis > 50,000/mm^3^ + no MI*	1			
*Leukocytosis > 50,000/mm^3^ + MI*	2			
AL myeloblastic *	2			
LVEF (%) mean ± SD	69.2 ± 2.7	ND		

NA: not applicable, AL: acute leukemia, MI: meningeal invasion, * AL myeloblastic: M2–M7, LVEF: left ventricular ejection fraction. ns: *p* > 0.05.

## Abbreviations

ALL	acute lymphoblastic leukemia
AML	acute myeloblastic leukemia
BMI	body mass index
CHO	carbohydrates
CPP	Comité de Protection des Personnes
HR	heart rate
IPAQ-Q	International Physical Activity Questionnaire for Adolescents
MET	metabolic equivalent of task
MFO	maximal fat oxidation
O_2_	oxygen
PA	physical activity
PAL	physical activity level
RER	respiratory exchange ratio
SD	standard derivation
VCO_2_	carbon dioxide volume
VO_2_	oxygen volume
W	watt
